# Dynamics of Neural Adjustments During Progressive Transitions to Unweighted and Reloaded Running

**DOI:** 10.1111/sms.70363

**Published:** 2026-08-24

**Authors:** Camille Fazzari, Robin Macchi, Rémy Casanova, Pascale Chavet, Jacques Duchateau, Caroline Nicol

**Affiliations:** ^1^ ISM, CNRS, Aix‐Marseille Université Marseille France; ^2^ Laboratoire Interuniversitaire de Biologie de la Motricité Université Jean Monnet Saint‐Etienne, Lyon 1, Université Savoie Mont Blanc Saint‐Etienne France; ^3^ Laboratoire de Biologie Appliquée Université Libre de Bruxelles Bruxelles Belgium

**Keywords:** body weight support, EMG activity, muscle synergy, perturbation, repeatability

## Abstract

The lower body positive pressure treadmill (LBPPT) enables running at different percentages of body weight. This study investigated how neural adjustments unfold during progressive transitions to unweighted and reloaded running, and assessed the repeatability of their dynamics across two successive exposures. Thirty‐eight men completed two runs (RUN1, RUN2) on a LBPPT. Each run comprised three stable phases at 100%, 60%, and 100% body weight (3 × 3 min), separated by progressive unweighting and reloading transitions (UNW_tr_ and RLD_tr_; 12 ± 2 s). Surface electromyographic (EMG) activity of 11 lower limb muscles was recorded. Root mean square EMG and muscle synergies were analyzed for each running cycle separately. In RUN1, some adjustments—including those in hamstring preactivation, as well as in *triceps surae* braking and push‐off activity—remained incomplete at the end of UNW_tr_. Moreover, a stabilizing muscle synergy was transiently recruited. In RUN2, however, these adjustments occurred more rapidly. In both runs, all neural adjustments were completed at the end of RLD_tr_. During UNW_tr_, runners encountered a novel sensorimotor context, where the gradual decrease in mechanical constraints interacted with the gradual increase in sensory and temporal constraints. Such context challenged the nervous system in RUN1, but was integrated more rapidly in RUN2, providing strong evidence of savings. In contrast, RLD_tr_ reinstated a familiar sensorimotor context, allowing for complete neural adjustments in both runs. These results highlight the relevance of unweighing and reloading transitions for evaluating and rehabilitating locomotor adaptability in clinical and athletic settings.

## 1 Introduction

The lower body positive pressure treadmill (LBPPT) consists of a flexible chamber sealed around the user's waist with a zip. It generates a lifting force that partially offsets the gravitational force, thereby allowing one to run at a wide range of effective body weights, from 100% down to 20% body weight. The LBPPT is primarily used in clinical settings to reduce osteoarticular and musculoskeletal strain following a lower‐limb injury or surgery. Beyond its clinical applications, this device provides a unique opportunity to investigate how neural adjustments—namely modifications in motor command and, consequently, in muscle activities—unfold during progressive transitions to unweighted and reloaded running.

Most studies using a LBPPT have focused on the stable phase of unweighted running, during which the effective body weight is kept constant. The earliest study reported an overall reduction in the surface electromyographic (EMG) activity of four lower limb muscles [1]. However, subsequent research revealed muscle‐ and phase‐dependent neural adjustments [2, 3, 4, 5]. Notably, hamstring activity remained unchanged during the stance phase [2, 3], or increased during the push‐off phase when running at 60% body weight [5]. Although these studies provide valuable information about adjustments in the activity of individual muscles, they do not establish whether these changes are accompanied by modifications in inter‐muscular coordination. To address this limitation, our recent study analyzed muscle synergies, defined as groups of muscles that receive a common neural input [6, 7]. The synergy involving the hamstrings differed between 100% and 60% body weight, confirming the increased contribution of this muscle group to the stance phase during unweighted running. Besides, this analysis emphasized the paradoxical nature of this condition. Reduced mechanical constraints allowed for more complex temporal components of muscle synergies, which are indicative of flexible locomotor control. In contrast, increased sensory and temporal constraints during the stance phase led to wider and delayed temporal components, reflecting the need for robust locomotor control [8]. Opposite neural adjustments have been reported during the stable phase of reloaded running, with minor or no aftereffects [4, 5, 8].

To date, only one study has examined the transition phases to unweighted and reloaded running, during which the effective body weight progressively decreases and increases, respectively [9]. This study reported a cycle‐to‐cycle reduction in the braking activity of the *soleus* and *vasti* muscles during a 15‐s transition from 100% to 60% body weight. Such decrease in EMG activity was positively correlated with a decrease in braking force. Yet, this study did not monitor hamstring activity, for which specific adjustments have since been identified [5, 8]. Furthermore, it overlooked the early stable phases of unweighted and reloaded running (i.e., the first running cycles immediately following the transitions). Therefore, it remains unclear whether runners continue to adjust beyond the end of the transitions. It is well‐established that prolonged exposure to a perturbation can attenuate its effects [10, 11]. For example, when running with elastics connecting the hips and ankles, the EMG activity of some lower limb muscles was found to initially increase, then gradually decrease over successive cycles, sometimes returning to unperturbed levels [11]. These limitations highlight the need to further investigate the dynamics of neural adjustments—not only during the transition phases, but also during the subsequent early stable phases.

In previous studies, we reported that the neural adjustments observed during the late stable phases of unweighted and reloaded running (i.e., last 40 s of a 3‐min run at constant body weight) were repeatable across two successive exposures [5, 8]. The only difference was a reduced EMG activity during the second exposure, irrespective of body weight, likely reflecting familiarization with LBPPT running [12]. However, repeated exposure to a perturbation generally results in faster adjustments than the initial exposure—a phenomenon known as ‘savings’ [13, 14]. For instance, when walking on a split‐belt treadmill, step length symmetry initially decreases, before being corrected over successive cycles; it recovers more rapidly upon re‐exposure [13]. Beyond faster adjustments, repeated exposure to a perturbation may also lead to refined adjustments. In particular, repeated exposure to gait‐slip perturbations has been reported to induce the recruitment of additional muscle synergies during the first exposures, through the fractionation of existing ones. Some of these synergies were no longer observed during subsequent exposures, suggesting more efficient locomotor control [15]. Taken together, these findings underscore the importance of examining how the dynamics of neural adjustments evolve during a second transition to unweighted and reloaded running.

Against this background, the aim of this study was twofold: (i) to characterize the dynamics of neural adjustments during progressive transition phases to unweighted and reloaded running, and (ii) to assess the repeatability of such dynamics across two successive exposures. We hypothesized that some neural adjustments would be incomplete at the end of the first unweighting transition. We also expected these adjustments to be achieved more rapidly during a second unweighting transition. Finally, we hypothesized that the adjustments would follow similar dynamics across the unweighting and reloading transitions in both runs.

## 2 Materials and Methods

### 2.1 Preamble

Our previous studies have examined the neural adjustments during the late stable phases of unweighted and reloaded running (i.e., the last 30 s and 60 running cycles of a 3‐min run at 60% and 100% body weight, respectively) [5, 8]. This research builds on the same participants, experimental protocol and recordings. Yet, it analyses entirely different data subsets, focusing on the dynamics of neural adjustments during the transition and early stable phases through a cycle‐by‐cycle analysis.

### 2.2 Participants

This study involved thirty‐eight physically active male students (age: 19 ± 1 years old, height: 177 ± 6 cm, mass: 69 ± 8 kg). All were free of musculoskeletal injuries and sensory disorders for at least 1 year. None of them had any prior experience of unweighted running. The procedures were approved by the National Ethics Committee for Research in Sports Sciences (reference no. CERSTAPS IRB00012476‐2021‐31‐03‐96). All participants gave written informed consent prior to the experiment, in accordance with the Declaration of Helsinki.

### 2.3 Experimental Protocol

**FIGURE 1:**
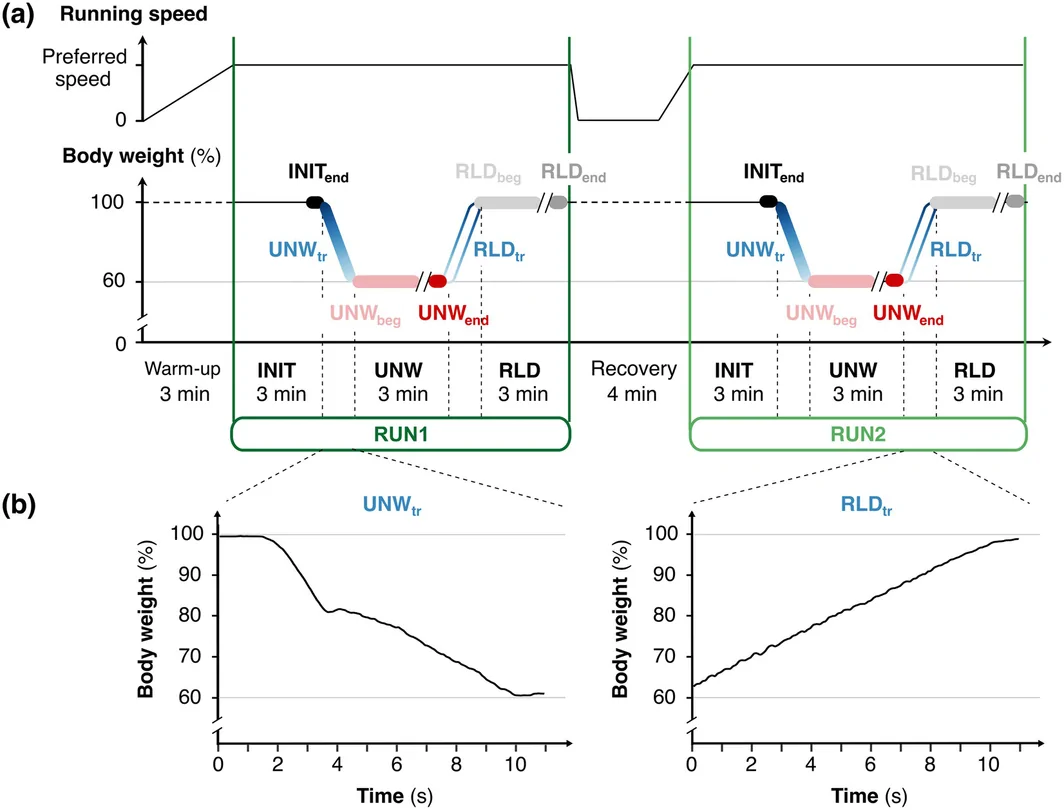
**Experimental design.** (a) Participants completed two runs (RUN1 in dark green, RUN2 in light green) on LBPPT. Each run included three stable phases, initial (INIT), unweighted (UNW) and reloaded (RLD) (3 min), separated by progressive unweighting and reloading transition phases (UNW_tr_ and RLD_tr_ in blue) (12 ± 2 s). The analysis included all running cycles during the transition phases (16 ± 4 cycles), as well as during both the early (first 150 cycles; UNW_beg_ in light red, RLD_beg_ in light gray) and the late (last 30 cycles; INIT_end_, in black; UNW_end_ in red; RLD_end_ in gray) stable phases. (b) The dynamics of the transitions was verified a posteriori for a representative participant. It was consistent with that previously reported [9].

Participants ran on a LBPPT (ViA400X AlterG, Fremont, CA, USA). More precisely, they performed a 3‐min warm‐up to gradually reach their preferred speed (3.0 ± 0.2 m/s, range: 2.8–3.3 m/s). This speed was self‐selected during a 15‐min familiarization run held 1 week earlier at 100% body weight. Subsequently, they completed two 9‐min runs (RUN1 and RUN2), separated by a 4‐min recovery period (3 min standing, 1 min walking). Each run consisted of three consecutive 3‐min stable phases: the initial (INIT) and reloaded (RLD) stable phases were performed at 100% body weight, while the unweighted (UNW) stable phase was performed at 60% body weight (Figure 1a). The stable phases were separated by progressive unweighting and reloading transitions (UNW_tr_ and RLD_tr_, respectively), which lasted 12 ± 2 s and included 16 ± 4 running cycles (Figure 1b).

### 2.4 Data Recordings

The vertical component of the ground reaction force was recorded using instrumented insoles (Loadsol, Novel, Munich, Germany; 100 Hz) placed in standardized running shoes (Run active, Kalenji). Each insole was divided into three zones: the rearfoot, medial forefoot, and lateral forefoot. The two forefoot zones were combined. Accelerations of the pelvis (L5 vertebra) and of the right forefoot (fifth metatarsal) were recorded using accelerometers (±16 g, 2000 Hz), and surface EMG activity was recorded using bipolar electrodes (2000 Hz). Both sensors were connected to the same acquisition system (miniWave COMETA, Milan, Italy). Specifically, the EMG signals were recorded from the following eleven muscles of the right lower limb: *Tibialis anterior* (TA), *gastrocnemius medialis* (GM), *gastrocnemius lateralis* (GL), *soleus* (SOL), *peroneus longus* (PL), *vastus medialis* (VM), *vastus lateralis* (VL), *rectus femoris* (RF), *biceps femoris* (BF), *semitendinosus‐semimembranosus* (ST‐SM), and *gluteus maximus* (GMax). Skin preparation and electrode placement followed the SENIAM recommendations, except that the skin was not abraded. The electrodes were reinforced with adhesive tape and covered with a self‐adherent elastic wrap, which proved effective in limiting their displacement despite repeated foot impacts. The force, accelerometric and EMG signals were synchronized using a ground impact.

### 2.5 Data Analysis

#### 2.5.1 Running Cycles Analyzed

The analysis included all running cycles during the transition phases (UNW_tr_ and RLD_tr_). It also covered the first 150 cycles of UNW and RLD corresponding to the early stable phases (UNW_beg_ C1‐150 and RLD_beg_ C1‐150), as well as the last 30 cycles of INIT, UNW and RLD corresponding to the late stable phases (INIT_end_ C1‐30, UNW_end_ C1‐30 and RLD_end_ C1‐30) (Figure 1a). Preliminary results revealed that all neural adjustments were completed by UNW_beg_ C150 and RLD_beg_ C150.

The number of running cycles within the transitions varied between participants due to differences in stride frequency. To standardize the analyses across participants, each transition was divided into eight intervals representing 12.5% of its total duration. The running cycles were assigned to such intervals based on their relative position within the transition. For example, if a participant completed 14 cycles, the first cycle corresponded to 0% of the transition, the fourteenth to 100%, and the intermediate were assigned proportionally. The variables were averaged within each interval, leading to 8 averaged cycles ($\bar{C}$) for each transition (UNW_tr_ $\bar{C}$1–$\bar{C}$8 and RLD_tr_ $\bar{C}$1–$\bar{C}$8). In total, the analyses included 812 cycles per participant.

#### 2.5.2 Biomechanical Adjustments

Touchdown and toe‐off were identified from the normal component of the ground reaction force using a 50 N threshold. The vertical acceleration of the pelvis was low‐pass filtered using a 4th order Butterworth zero‐phase filter (cut‐off frequency: 10 Hz), and then double integrated. The minimum vertical position of the pelvis indicated the end of the braking phase. The braking time, push‐off time, stance time, swing time and stride time were computed. The active peak force, and the mean vertical force during the braking and push‐off phases were calculated. Moreover, the mean vertical force under the forefoot and rearfoot were calculated throughout the stance phase (${\bar{F}}_{\text{forefoot}}$ and ${\bar{F}}_{\text{rearfoot}}$). These were used to compute the loading index, as defined in Equation (1).

(1) $$\text{Loading index}=\left({\bar{F}}_{\text{forefoot}}-{\bar{F}}_{\text{rearfoot}}\right)/\left({\bar{F}}_{\text{forefoot}}+{\bar{F}}_{\text{rearfoot}}\right)$$

According to this definition, the loading index is zero when the forefoot and rearfoot are loaded equally. It is positive when the forefoot is loaded more than the rearfoot, and negative when the opposite occurs.

#### 2.5.3 Neural Adjustments Based on RMS EMG

The raw EMG signals were first band‐pass filtered using a 4th order Butterworth zero‐phase filter (cut‐off frequencies: 10–500 Hz), full‐wave rectified, and low‐pass filtered (cutoff frequency: 75 Hz) using a critically damped filter [16]. They were shifted to zero by subtracting their minimum value. They were normalized to the average of the maximum EMG values recorded within each running cycle at INIT_end_ in RUN1.

The root mean square of the pre‐processed EMG signals was calculated separately for each phase of the stretch‐shortening cycle (preactivation, braking, and push‐off). The preactivation phase was defined as the 100 ms preceding touchdown.

#### 2.5.4 Neural Adjustments Based on Muscle Synergies

The band‐pass filtered EMG signals were subsequently high‐pass filtered (cut‐off frequency: 50 Hz), full‐wave rectified, and low‐pass filtered using a 4th order IIR Butterworth zero‐phase filter (cut‐off frequency: 20 Hz). They were offset to zero by subtracting their minimum value, as is commonly done prior to muscle synergy extraction. Each cycle was amplitude‐normalized to its maximum EMG value. Finally, it was time‐normalized to 200 time points, with 100 points assigned to the stance phase and 100 points to the swing phase [17].

##### 2.5.4.1 Extraction of Muscle Synergies

Muscle synergies were extracted using a R script (v3.6.3, R Foundation for Statistical Computing, Vienna, Austria), based on the R package “musclesyneRgies” [18]. For each running cycle separately, the pre‐processed EMG signals were arranged into a matrix *V* with dimensions *m × p*, where *m =* 11 (one row per muscle) and *p* = 200 (one column per time point). This matrix was factorized using the Gaussian non‐negative matrix factorization (NMF) algorithm, as shown in Equation (2).

(2) $$V\approx V_{R}=\mathrm{MP}$$

The reconstructed matrix $V_{R}$ approximates the original matrix *V* as the product of two non‐negative matrices. *M* contains the motor modules (i.e., the time‐invariant muscle contributions to a synergy), while *P* contains the motor primitives (i.e., the time‐varying activation of a synergy). The scalar *r* represents the factorization rank (i.e., the minimum number of muscle synergies required to satisfactorily reconstruct *V*).

The matrices *M* and *P* were initialized with random non‐negative values. They were updated iteratively according to the rules presented in Equations (3) and (4).

$$\left\{\begin{array}{cc} P_{i+1}=P_{i}⊙\frac{M_{i}^{T}V}{M_{i}^{T}M_{i}P_{i}} & \left(3\right)\\ M_{i+1}=M_{i}⊙\frac{V{\left(P_{i+1}\right)}^{T}}{M_{i}P_{i+1}{\left(P_{i+1}\right)}^{T}} & \left(4\right) \end{array}\right.$$

where i∈ℕ is the iteration index, *T* the transpose matrix, and ʘ the element‐wise product.

The reconstruction quality was assessed by calculating the coefficient of determination (*R* ^2^) between *V* and *V*_*R*_. The algorithm was stopped when the relative change in *R* ^2^ fell below 0.01% across 20 consecutive iterations [17, 19].

The matrix *V* was factorized for *r* ∈ {1, 2…,8}. The upper limit was set to 75% of the monitored muscles to ensure dimensionality reduction. The NMF algorithm was applied five times for each candidate rank, with *M* and *P* reinitialized each time to avoid convergence to a suboptimal local minima. The solution yielding the highest *R* ^2^ was retained. A linear regression was fitted to the *R* ^2^ vs. *r* curve. The mean square error between the regression line and the empirical curve was iteratively recalculated. The leftmost point was excluded at each iteration, until two points remained or the mean square error fell below 10^−4^. The optimal factorization rank corresponded to the abscissa of the leftmost remaining point [17].

##### 2.5.4.2 Specificity of Extracting Muscle Synergies for Individual Cycles

While many previous studies have extracted muscle synergies from concatenated running cycles [20, 21] the present study extracted muscle synergies from each running cycle separately. This approach allowed the factorization axes (i.e., the motor modules and motor primitives) and the factorization rank (i.e., the number of synergies) to fluctuate between the cycles, thereby capturing subtle adjustments. Yet, it increased the risk of identifying noise‐driven synergies with limited functional relevance. To mitigate this limitation, the extraction procedure was repeated 20 times for each cycle [22]. Only the synergies that could be classified (i.e., identified as part of a stable cluster across multiple cycles) were retained and averaged.

##### 2.5.4.3 Classification of Muscle Synergies

For each running cycle separately, the motor modules obtained from all participants were pooled and classified using a *k*‐means algorithm, for a number of clusters ranging from 1 to 11. The classification was repeated 20 times for each number of clusters, using different initial centroids each time. The solution that minimized intra‐cluster variance was retained. A linear regression was fitted to the intra‐cluster variance versus number of clusters curve. The optimal number of clusters was determined using the same procedure as for selecting the factorization rank. The motor primitives were then classified by imposing the same number of clusters selected for the motor modules. Both classifications were compared: When the clusters assigned to a motor module and its corresponding motor primitive were discordant, the synergy was classified as ‘combined’. It was excluded from further analysis.

##### 2.5.4.4 Characterization of Motor Modules

To compare motor modules across running cycles, we calculated their cosine similarity relative to the mean motor modules obtained at INIT_end_ in RUN1. Let $M_{\;j}^{A}$ denote the mean motor module of the j^th^ synergy extracted at INIT_end_ in RUN1, and $M_{\;j}^{B}$ the motor module of the j^th^ synergy for each analyzed running cycle. The cosine similarity was calculated following Equation (5), where *m* corresponds to the number of muscles.

(5) $$\text{cosine similarity}\;\left(M_{\;j}^{A},M_{\;j}^{B}\right)=\frac{M_{\;j}^{A}.M_{\;j}^{B}}{\left\|M_{\;j}^{A}\right\|.\left\|M_{\;j}^{B}\right\|}=\frac{\sum_{i=1}^{m}{\left(M_{\;j}^{A}\right)}_{i}.{\left(M_{\;j}^{B}\right)}_{i}}{\sqrt{\sum_{i=1}^{m}{{\left(M_{\;j}^{A}\right)}_{i}}^{2}}\sqrt{\sum_{i=1}^{m}{{\left(M_{\;j}^{B}\right)}_{i}}^{2}}}$$

The cosine similarity typically ranges from −1 to 1. Notably, it is 0 when the motor modules are orthogonal (unrelated), and 1 when they are identical [22]. Since the motor modules are inherently non‐negative, the cosine similarity was constrained to the interval [0; 1].

##### 2.5.4.5 Characterization of Motor Primitives

To compare motor primitives across running cycles, we calculated their center of activity (CoA), full width at half maximum (FWHM), and Higuchi's fractal dimension (HFD).

The CoA indicates the timing of a synergy. Let $P_{j}\left(t\right)=\left[P_{j}\left(1\right),P_{j}\left(2\right),\ldots P_{j}\left(200\right)\right]$ be the primitive of the j^th^ synergy. Each time point was mapped to an angle $\theta \left(t\right)=\left(2\pi \left(t-1\right)\right)/200.$ The CoA was obtained according to Equations (6)–(8).

$$\left\{\begin{array}{cc} A=\sum_{t=1}^{n}\cos\left(\theta \left(t\right)\right)\times P_{j}\left(t\right) & \left(6\right)\\ B=\sum_{t=1}^{n}\sin\left(\theta \left(t\right)\right)\times P_{j}\left(t\right) & \left(7\right)\\ \mathrm{CoA}=\arctan\left(B/A\right) & \left(8\right) \end{array}\right.$$

where *n* = 200 is the number of time points.

The CoA ranges from 0° to 360°, with 0°–180° corresponding to the stance phase and 180°–360° to the swing phase [23]. For ease of interpretation, the CoA was converted into normalized time points (0° = 1; 360° = 200 time points), corresponding to the normalized running cycle.

The FWHM indicates the duration of a synergy. The primitive was shifted to zero by subtracting its minimum value, and normalized to its maximum value. The FWHM was calculated as the number of points exceeding half the maximum of the primitive (i.e., exceeding 0.5) [23].

Finally, the HFD quantifies the local complexity (or “roughness”) of a motor primitive. Let $P_{j}\left(t\right)=\left[P_{j}\left(1\right),P_{j}\left(2\right),\ldots P_{j}\left(200\right)\right]$ be the primitive of the j^th^ synergy. It was subsampled to construct k subseries, as shown in Equation (9).

(9) $${\left(P_{j}\right)}_{k}^{t_{0}}=\left[P_{j}\left(t_{0}\right),\;P_{j}\left(t_{0}+k\right),\;P_{j}\left(t_{0}+2k\right),\;\ldots ,\;P_{j}\left(t_{0}+\left\lfloor \frac{n-t_{0}}{k}\right\rfloor k\right)\right]$$

where *n* = 200 the number of time points, ⌊·⌋ denotes the integer (lower) part, $t_{0}\in \left\{1,2,\ldots k\right\}$ the starting point, and $k\in \left\{1,2,\ldots ,k_{\max}\right\}$ the subsampling rate.

The non‐Euclidean length of each sub‐series was then calculated according to (Equation 10).

(10) $$L_{t_{0}}\left(k\right)=\frac{1}{k}\;\left[\frac{n-1}{\left\lfloor \frac{n-t_{0}}{k}\right\rfloor \cdot k}\sum_{i=1}^{\left\lfloor \frac{n-t_{0}}{k}\right\rfloor \;}\left|P_{j}\left(t_{0}+\mathrm{ik}\right)-P_{j}\left(t_{0}+\left(i-1\right)k\right)\right|\right]$$

The non‐Euclidean length of the motor primitive was calculated as shown in Equation (11).

(11) $$L\left(k\right)=\frac{1}{k}\;\sum_{t_{0}=1}^{k}L_{t_{0}}\left(k\right)$$

The HFD corresponded to the slope of the linear regression of $\log\left(L\left(k\right)\right)$ vs. $\log\left(1/k\right)$ for $k\in \left\{1,2,\ldots ,k_{\max}\right\}$. It was obtained using the least squares method. The value of $k_{\max}$ was determined by repeating the above procedure for k∈ {1, 2, 3, … 300}, and selecting the highest value of *k* for which the $\log\left(L\left(k\right)\right)$ versus $\ln\left(1/k\right)$ relationship remained linear [24]. In our data, this corresponded to $k_{\max}$ = 10.

The HFD ranges from 1 to 2, with values near to 1 indicating low roughness (e.g., linear or low frequency sinusoidal signals), and values near to 2 indicating high roughness (e.g., white Gaussian noise).

##### 2.5.4.6 Fractionation of Muscle Synergies

A visual inspection suggested that some synergies identified at INIT_end_ in RUN1 may be fractionated at UNW_tr_ $\bar{C}$1 and UNW_tr_ $\bar{C}$2 in RUN1. Let $M^{A}=$ {$M_{\;1}^{A},M_{\;2}^{A},\ldots ,M_{\;r}^{A}$} be the motor modules at INIT_end_ in RUN1, and $M^{B}$ the motor modules at UNW_tr_ $\bar{C}$1 (or $\bar{C}$2) in RUN1, with *r* being the factorization rank. $P^{A}$ and $P^{B}$ are the corresponding motor primitives. We examined whether the motor modules (primitives) extracted at INIT_end_ in RUN1 could be reconstructed as a linear combination of those extracted at UNW_tr_ $\bar{C}$1 (or $\bar{C}$2) in RUN1, according to Equations (12) and (13).

(12) $$M_{\;j}^{A}={\left(M_{\;j}^{A}\right)}_{R}\cdot \sum_{i=1}^{n_{f}}\alpha_{i}M_{\;i}^{B}$$

(13) $$P_{\;j}^{A}={\left(P_{\;j}^{A}\right)}_{R}\cdot \sum_{i=1}^{n_{f}}\beta_{i}P_{\;i}^{B}$$

where $n^{f}$ is the number of muscle synergies from UNW_tr_ $\bar{C}$1 (or $\bar{C}$2) in RUN1 necessary to reconstruct the j^th^ synergy observed at INIT_end_ in RUN1, and $\alpha_{i}$ (or *β*_*i*_) are non‐negative coefficients.

The coefficients $\alpha_{i}$ (or *β*_*i*_) were estimated via non‐negative least squares (R package *nnls*). A synergy was considered to be fractionated if at least two synergies contributed to its reconstruction ($n^{f}\geq 2$), each contributing synergy had a weight $\alpha_{j}\;\left(\text{or}\;\beta_{i}\right)>0.2$, and the cosine similarity between the original and the reconstructed motor modules (primitives) was higher than 0.8 [25, 26].

#### 2.5.5 Statistics

For each variable, a linear mixed model was fitted using restricted maximum likelihood estimation (R package *LmerTest*) [27]. Running cycle (406 levels) and Run (2 levels) were included as fixed effects, while the intercept of the participant was included as a random effect, and preferred speed as a covariate. The significance of the random intercept was tested. Fixed and interaction effects were selected using a backward selection method, based on likelihood ratio tests of nested models. Residual normality was assessed visually using Q–Q plots, and no severe violation was observed. An analysis of variance (ANOVA) was performed on the selected model, with degrees of freedom estimated using the Satterthwaite method. When significant main or interaction effects were identified, post hoc tests were conducted with *p*‐values adjusted using either Holm's method or Benjamini‐Hochberg's method. The latter was used when the number of pairwise comparisons exceeded twenty [28]. Specifically, post hoc tests were used to:

- Assess the stability of the adjustments during the last 30 cycles of INIT, UNW and RLD (INIT_end_, UNW_end_ and RLD_end_);
- Evaluate the adjustments between these late stable phases, enabling direct comparison with previous analyses based on concatenated cycles [5, 8];
- Characterize the dynamics of adjustments during the transition phases (UNW_tr_ and RLD_tr_) and early stable phases (UNW_beg_ and RLD_beg_). Each cycle within UNW_tr_ $\bar{C}$1–$\bar{C}$8 and UNW_beg_ C1–150 was compared to INIT_end_ (initial state) and UNW_end_ (final state). Similarly, each cycle within RLD_tr_ $\bar{C}$1–$\bar{C}$8 and RLD_beg_ C1‐150 was compared to both UNW_end_ (initial state) and RLD_end_ (final state). To identify consistent rather than isolated adjustments, a significant interval began when at least three consecutive running cycles differed significantly from the reference, and ended when at least three consecutive cycles no longer did. The onset of adjustment corresponded to first cycle of the first significant interval differing from the initial state. The completion corresponded to the last cycle of the last interval differing from the final state. For all significant comparisons, the effect size (ES) was calculated based on Cohen's d. Trivial effects (ES < 0.2) were not reported.

Spearman's correlation tests were conducted for each participant and muscle to assess the relationship between the changes in muscle activity and mean vertical force during UNW_tr_, in both the braking and push‐off phases. Chi‐square tests were then used to compare the proportions of participants showing significant positive and significant negative correlations between muscle activity and mean vertical force.

All statistics were conducted using a custom R script, with significance set at *α* = 0.05. The results are presented as mean ± standard deviation.

## 3 Results

For all variables, no significant differences were found within the 30 running cycles constituting the late stable phases (INIT_end_, UNW_end_, and RLD_end_; *p* > 0.05 for all pairwise comparisons), validating their use as references to determine the onset and completion of adjustments during the transition and subsequent early stable phases. The adjustments observed between the late stable phases (UNW_end_ vs. INIT_end_, RLD_end_ vs. UNW_end_, RLD_end_ vs. INIT_end_) were highly consistent with those previously identified using an averaged cycle analysis [5, 8], reinforcing confidence in our cycle‐by‐cycle analysis.

### 3.1 Biomechanical Adjustments

**FIGURE 2:**
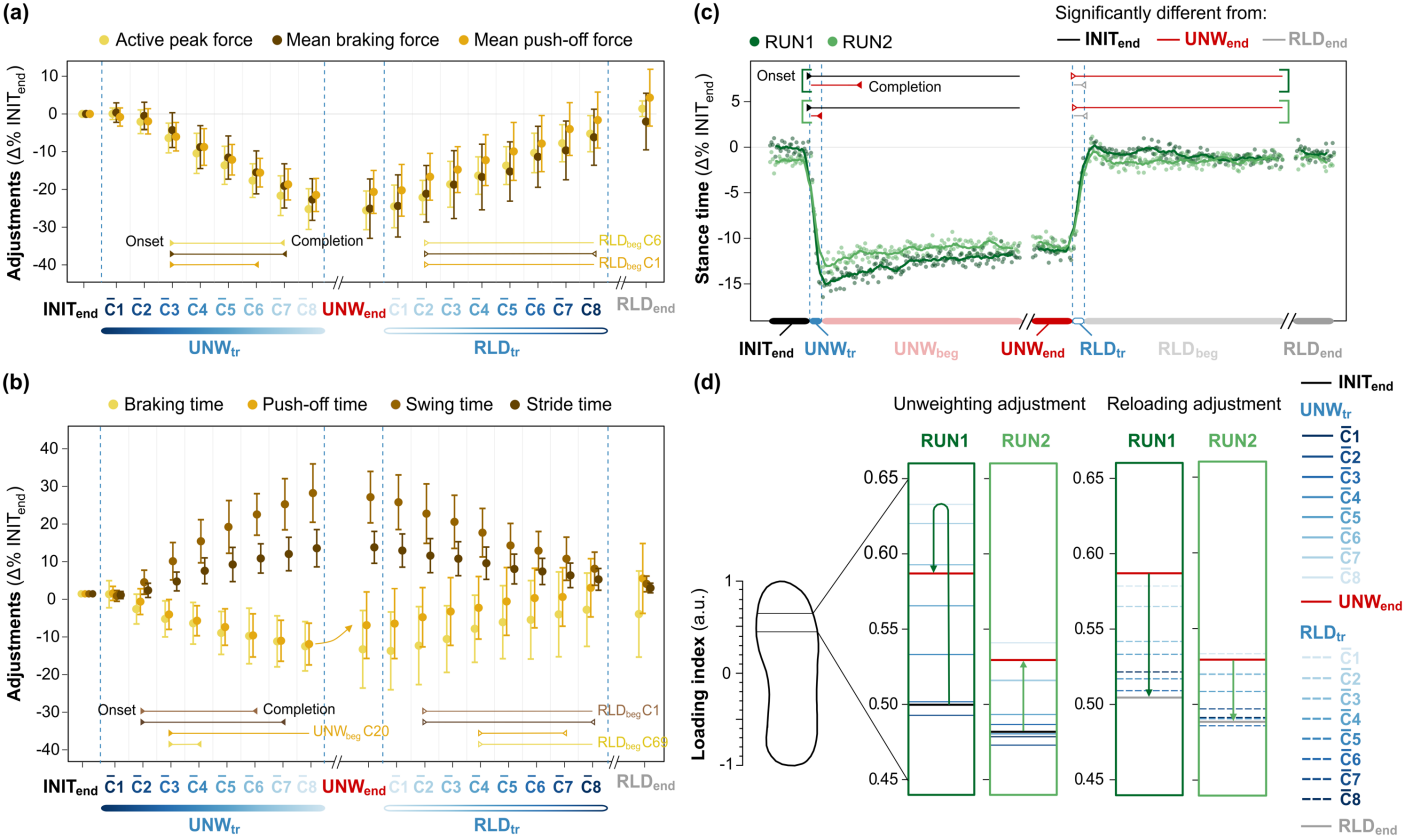
**Dynamics of biomechanical adjustments.** (a) Adjustments in active peak force, mean braking force and mean push‐off force. They are expressed as percentage changes (mean ± SD) relative to INIT_end_. They are shown for each cycle during the transitions (UNW_tr_ $\bar{C}$1–$\bar{C}$8 and RLD_tr_ $\bar{C}$1–$\bar{C}$8, in blue), and averaged over the 30 cycles constituting the late stable phases (INIT_end_ in black, UNW_end_ in red and RLD_end_ in gray). (b) Adjustments in braking, push‐off, swing and stride times, shown in the same format as in (a). (c) Adjustments in stance time are shown for each cycle across the transitions, early stable phases (UNW_beg_ in light red, RLD_beg_ in light gray), and late stable phases. They are presented separately for RUN1 (dark green) and RUN2 (light green), due to a Cycle × Run interaction, and smoothed with a 10‐cycle centered moving average. In panels (a–c), arrowheads indicate the onset and completion of the adjustments. In panels (a, b), when completion occurred after the transition, the corresponding cycle is specified. (d) The loading index (without unit) is shown for the same cycles as in (a, b) but separately for the two runs. Arrows indicate the dynamics of adjustments.

The kinetic and spatiotemporal adjustments are illustrated in Figure 2. A significant Cycle effect was found for active peak force (*F*_(405, 28896)_ = 434), as well as for mean vertical braking and push‐off forces (*F*_(405, 28896)_ = 98 and *F*_(405, 28896)_ = 133, respectively) (*p* < 0.001) (Figure 2a). These forces decreased progressively during UNW_tr_ and increased during RLD_tr_.

There was also a Cycle effect for braking time (*F*_(405, 28896)_ = 16), push‐off time (*F*_(405, 28896)_ = 21), swing time (*F*_(405, 28896)_ = 268) and stride time (*F*_(405, 28896)_ = 162) (*p* < 0.001) (Figure 2b). During UNW_tr_, braking and push‐off times decreased, whereas swing and stride times increased gradually. Push‐off time exhibited a transient larger decrease than thereafter (called excessive decrease) between UNW_tr_ $\bar{C}$7‐UNW_beg_ C20 (ES = 0.23 ± 0.03). During RLD_tr_, braking and push‐off times increased, while swing and stride times decreased progressively. Braking time showed a transient excessive increase between RLD_beg_ C5–C18, RLD_beg_ C35–C56 and RLD_beg_ C63–C69 (ES = 0.23 ± 0.02).

Stance time showed a Cycle effect (*F*_(405, 28896)_ = 113, *p* < 0.001), along with a Cycle × Run interaction (*F*_(405, 28896)_ = 1, *p* < 0.001) (Figure 2c). During UNW_tr_, it shortened gradually, but the adjustment dynamics differed between runs. In RUN1, stance time exhibited a transient excessive decrease between UNW_tr_ $\bar{C}$7‐UNW_beg_ $\bar{C}$26 (ES = 0.29 ± 0.07). In RUN2, the adjustment occurred earlier, without overshoot. During RLD_tr_, stance time lengthened progressively in both runs.

Finally, a Cycle effect was identified for the loading index (*F*_(405, 28896)_ = 12, *p* < 0.001), along with a Cycle × Run interaction (*F*_(405, 28896)_ = 2, *p* < 0.001) (Figure 2d). During UNW_tr_, this index increased gradually, indicating further forefoot loading, but the adjustment dynamics differed between the two runs. In RUN1, it showed a transient excessive increase between UNW_tr_ $\bar{C}$8‐UNW_beg_ C17 (ES = 0.33 ± 0.06). In RUN2, the adjustment was completed almost immediately, without overshoot. Notably, the loading index reached a higher value at UNW_end_ in RUN1 than in RUN2 (*p* < 0.001, ES = 0.36). During RLD_tr_, it declined progressively in both runs.

### 3.2 Neural Adjustments Based on RMS EMG

**FIGURE 3:**
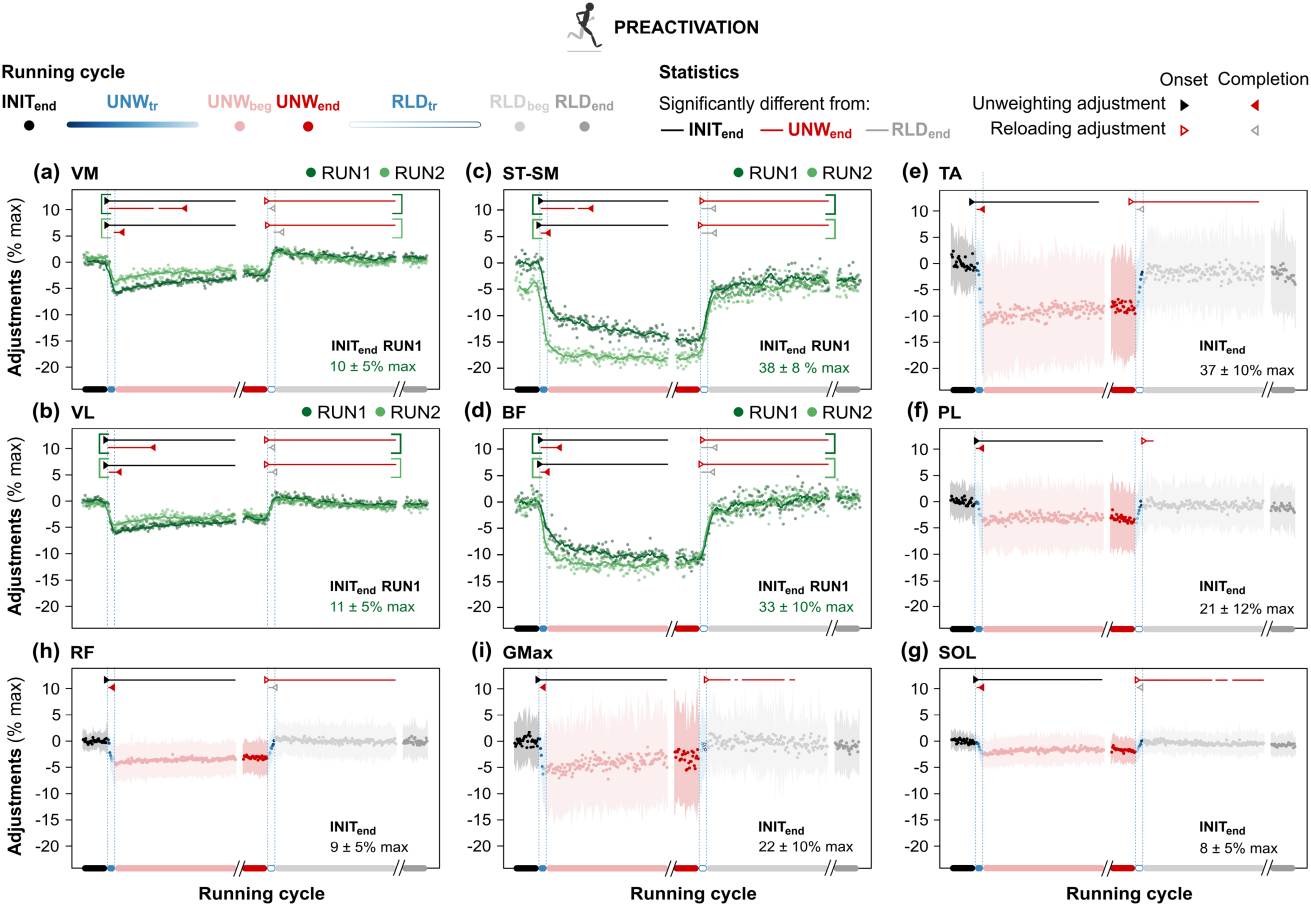
**Dynamics of EMG activity adjustments during the preactivation** **phase**. Adjustments in the preactivation of the (a) *vastus medialis* (VM), (b) *vastus lateralis* (VL), (c) *semitendinosus‐semimembranosus* (ST‐SM), (d) *biceps femoris* (BF), (e) *tibialis anterior* (TA), (f) *peroneus longus* (PL), (g) *soleus*, (h) *rectus femoris* (RF), and (i) *gluteus maximus* (GMax). They are expressed as absolute changes from INIT_end_ in RUN1 or INIT_end_ averaged across both runs, with the corresponding reference value shown in the lower right‐hand corner. In panels (a–d), the adjustments (mean ± SD) are shown for each cycle during the transitions (UNW_tr_ and RLD_tr_, in blue), early stable phases (UNW_beg_ in light red, RLD_beg_ in light gray) and late stable phases (INIT_end_ in black, UNW_end_ in red and RLD_end_ in gray). They are presented separately for RUN1 (dark green) and RUN2 (light green), due to a Cycle × Run interaction, and smoothed with a 10‐cycle moving average. In panels (e–i), the adjustments are shown for the same cycles, but averaged over both runs. Horizontal bars indicate cycles that differ significantly from INIT_end_ (black), UNW_end_ (red) and RLD_end_ (gray). Arrowheads indicate the onset and completion of the adjustments.

**FIGURE 4:**
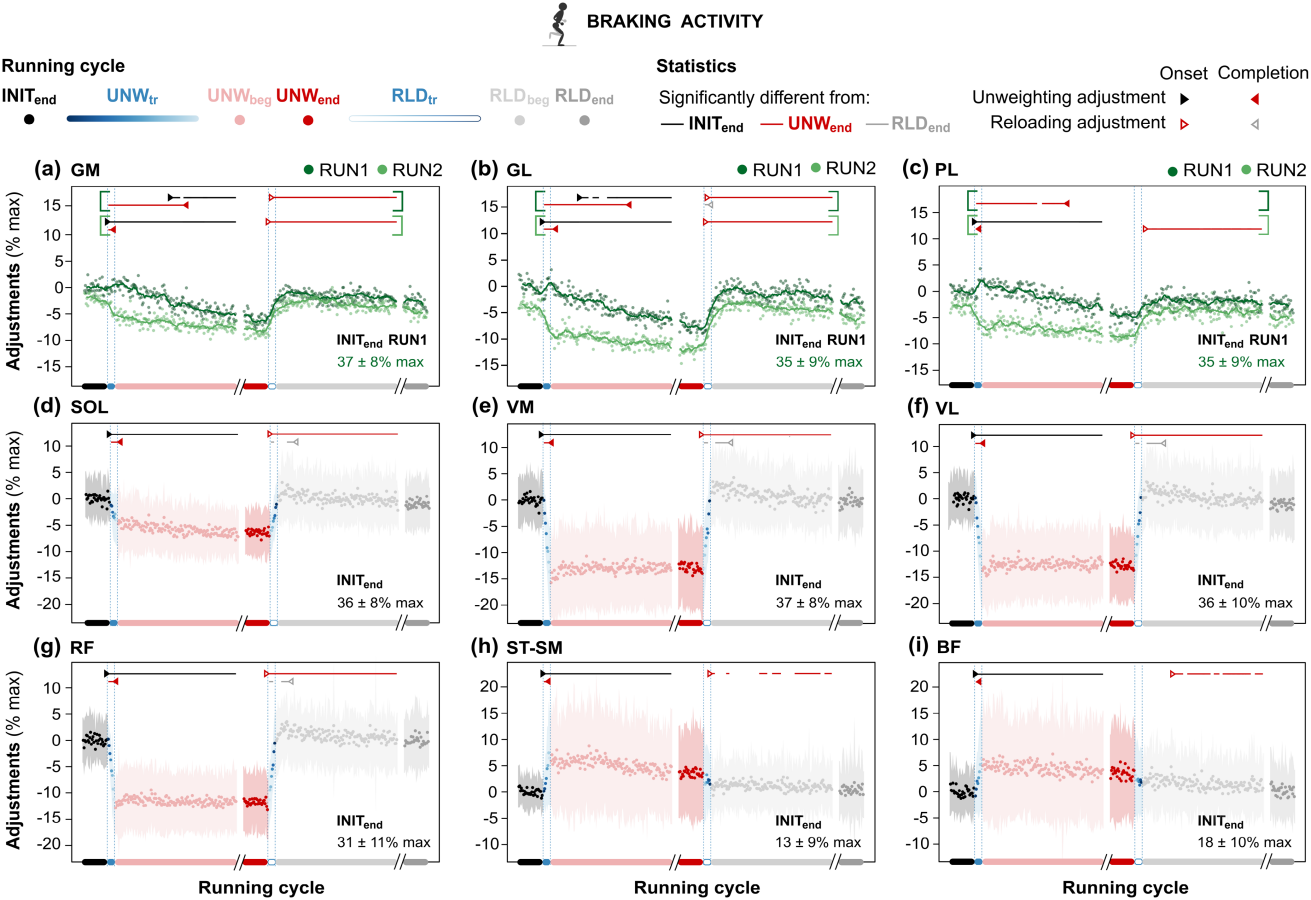
**Dynamics of EMG activity adjustments during the braking** **phase**. Adjustments in the braking activity of the: (a) *gastrocnemius medialis* (GM), (b) *gastrocnemius lateralis* (GL), (c) *peroneus longus* (PL), (d) *soleus* (SOL), (e) *vastus medialis* (VM), (f) *vastus lateralis* (VL), (g) *rectus femoris* (RF), (h) *semitendinosus‐semimembranosus* (ST‐SM), and (i) *biceps femoris* (BF). They are expressed as absolute changes from INIT_end_ in RUN1 or INIT_end_ averaged across both runs, with the corresponding reference value shown in the lower right‐hand corner. In panels (a–c), the adjustments (mean ± SD) are shown for each cycle during the transitions (UNW_tr_ and RLD_tr_, in blue), early stable phases (UNW_beg_ in light red, RLD_beg_ in light gray) and late stable phases (INIT_end_ in black, UNW_end_ in red and RLD_end_ in gray). They are presented separately for RUN1 (dark green) and RUN2 (light green), due to a Cycle × Run interaction, and smoothed with a 10‐cycle moving average. In panels (d–i), the adjustments are shown for the same cycles, but averaged over both runs. Horizontal bars indicate cycles that differ significantly from INIT_end_ (black), UNW_end_ (red) and RLD_end_ (gray). Arrowheads indicate the onset and completion of the adjustments.

**FIGURE 5:**
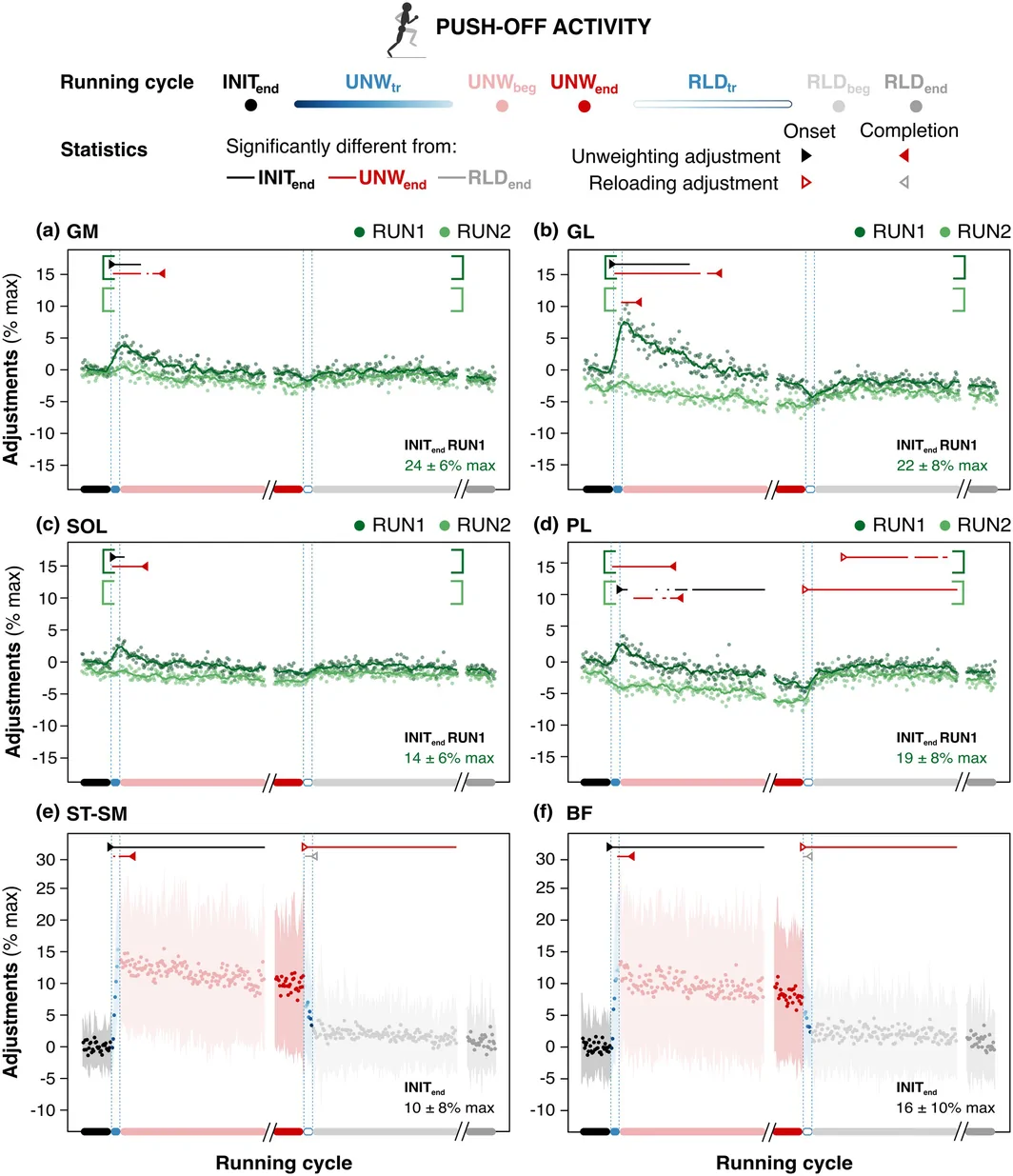
**Dynamics of EMG activity adjustments during the push‐off** **phase**. Adjustments are shown for the push‐off activity of the following muscles: (a) *gastrocnemius medialis* (GM), (b) *gastrocnemius lateralis* (GL), (c) *soleus* (SOL), (d) *peroneus longus* (PL), (e) *semitendinosus‐semimembranosus* (ST‐SM), and (f) *biceps femoris* (BF). They are expressed as absolute changes from INIT_end_ in RUN1 or INIT_end_ averaged across both runs, with the corresponding reference value shown in the lower right‐hand corner. In panels (a–d), the adjustments (mean ± SD) are shown for each cycle during the transitions (UNW_tr_ and RLD_tr_, in blue), early stable phases (UNW_beg_ in light red, RLD_beg_ in light gray) and late stable phases (INIT_end_ in black, UNW_end_ in red and RLD_end_ in gray). They are presented separately for RUN1 (dark green) and RUN2 (light green), due to a Cycle × Run interaction, and smoothed with a 10‐cycle moving average. In panels (e, f), the adjustments are shown for the same cycles, but averaged over both runs. Horizontal bars indicate cycles that differ significantly from INIT_end_ (black), UNW_end_ (red) and RLD_end_ (gray). Arrowheads indicate the onset and completion of the adjustments.

The adjustments in RMS EMG are presented for each phase of the stretch‐shortening cycle: Preactivation, braking, and push‐off (Figures 3, 4, 5).

#### 3.2.1 Preactivation

Both *vasti* and hamstring preactivation showed a Cycle effect (VM and VL: *F*_(405, 28896)_ = 21; ST‐SM: *F*_(405, 28896)_ = 34, BF: *F*_(405, 28896)_ = 32; *p* < 0.001), along with a Cycle × Run interaction (VM, VL, ST‐SM: *F*_(405, 28896)_ = 2, BF: *F*_(405, 28896)_ = 1; *p* < 0.001) (Figure 3a–d). During UNW_tr_, they decreased progressively, though the adjustment dynamics differed between runs. In RUN1, *vasti* preactivation showed a transient excessive decrease: for VM, between UNW_tr_ $\bar{C}$4‐UNW_beg_ C84 (ES = 0.44 ± 0.12); and for VL, between UNW_tr_ $\bar{C}$6‐UNW_beg_ C48 (ES = 0.41 ± 0.08). In turn, hamstring preactivation showed a transient insufficient decrease: for ST‐SM, between UNW_tr_ $\bar{C}$1‐UNW_beg_ C50 (ES = 0.54 ± 0.29); and for BF, between UNW_tr_ $\bar{C}$1‐UNW_beg_ C11 (ES = 0.71 ± 0.28). In RUN2, these adjustments were completed as soon as UNW_beg_ C4 for VM, UNW_beg_ C2 for VL, UNW_tr_ $\bar{C}$6 for ST‐SM, and UNW_tr_ $\bar{C}$5 for BF, without over‐ or undershoot. During RLD_tr_, *vasti* and hamstring preactivation increased gradually in both runs.

A Cycle effect was found for the preactivation of the TA (*F*_(405, 25832)_ = 12), PL (*F*_(405, 28896)_ = 4), SOL (*F*_(405, 28896)_ = 6), RF (*F*_(405, 28081)_ = 27) and GMax (*F*_(405, 27274)_ = 4) (*p* < 0.001), without a Cycle × Run interaction (Figure 3e–i). During UNW_tr_, the preactivation of these muscles decreased progressively. Conversely, during RLD_tr_, TA, SOL, RF and GMax preactivation increased, and PL preactivation exhibited only sporadic adjustments. The reloading adjustment in GMax preactivation showed no completion, as it was already not significantly different from RLD_end_ at RLD_tr_ $\bar{C}$1. This was probably due to an increasing trend during UNW_beg_. Although g*astrocnemii* preactivation showed a Cycle effect (GM: *F*_(405, 28085)_ = 2, GL: *F*_(405, 28896)_ = 2; *p* < 0.001), post hoc analyses revealed only sporadic adjustments during either UNW_tr_ or RLD_tr_.

#### 3.2.2 Braking Activity

The braking activity of the *gastrocnemii* and PL showed a Cycle effect (GM: *F*_(405, 28085)_ = 5, GL: *F*_(405, 28896)_ = 9; PL: *F*_(405, 28896)_ = 2, *p* < 0.001), along with a Cycle × Run interaction (GM: *F*_(405, 28085)_ = 2, GL: *F*_(405, 28896)_ = 2, PL: *F*_(405, 28896)_ = 2; *p* < 0.001) (Figure 4a–c). Their unweighting adjustment dynamics differed between runs. In RUN1, *gastrocnemii* and PL braking activity remained unchanged during UNW_tr_, despite an apparent increase (all post hoc *p*‐values > 0.05; GM: 0.25–0.95, GL: 0.06–0.80, and PL: 0.34–0.92). Their activity declined progressively during UNW_beg_, the adjustment being completed at UNW_beg_ C84 for GM, C93 for GL, and C102 for PL. In RUN2, the adjustment was completed as soon as UNW_tr_ $\bar{C}$3 for GM, UNW_beg_ C2 for GL, UNW_tr_ $\bar{C}$1 for PL. During RLD_tr_, *gastrocnemii* and PL braking activity increased in both runs. No completion of these reloading adjustments could be identified, since the braking activity was already not significantly different from RLD_end_ at RLD_tr_ $\bar{C}$1.

We also found a Cycle effect for the braking activity of the SOL (*F*_(405, 28896)_ = 14), *quadriceps* muscles (VM: *F*_(405, 28896)_ = 52, VL: *F*_(405, 28896)_ = 47, RF: *F*_(405, 28084)_ = 57), and hamstring muscles (ST‐STM: *F*_(405, 28896)_ = 6, BF: *F*_(405, 28896)_ = 4) (*p* < 0.001) (Figure 4d–i). During UNW_tr_, SOL and *quadriceps* braking activity decreased, whereas hamstring braking activity increased progressively. Conversely, during RLD_tr_, SOL and *quadriceps* braking activity increased, whereas hamstring braking activity decreased. The braking activity of several muscles showed a transient excessive increase during RLD_beg_: for SOL, between C12–C19 (ES = 0.34 ± 0.05); for VM, between C6–C29 (ES = 0.35 ± 0.05); for VL, between C6–C23 (ES = 0.33 ± 0.06); and for RF, between C7–C15 (ES = 0.27 ± 0.05). The reloading adjustment in hamstring braking activity showed no completion, as it was already not significantly different from RLD_end_ at RLD_tr_ $\bar{C}$1. Although a Cycle effect was found for the TA (*F*_(405, 25832)_ = 2) and GMax (*F*_(405, 27274)_ = 1) (*p* < 0.001), post hoc analyses revealed only sporadic adjustments during either UNW_tr_ or RLD_tr_.

#### 3.2.3 Push‐Off Activity

The push‐off activity of the *triceps surae* and PL showed a Cycle effect (GM: *F*_(405, 28085)_ = 2, GL: *F*_(405, 28896)_ = 5, SOL: *F*_(405, 28896)_ = 2; PL: *F*_(405, 28896)_ = 3; *p* < 0.001) and a Cycle × Run interaction (GM: *F*_(405, 28085)_ = 1, GL: *F*_(405, 28896)_ = 3, SOL: *F*_(405, 28896)_ = 1, PL: *F*_(405, 28896)_ = 2; *p* < 0.001) (Figure 5a–d). Their unweighting adjustment dynamics differed between runs. In RUN1, *triceps surae* and PL push‐off activity increased during UNW_tr_, but decreased progressively during UNW_beg_. The adjustment was completed at UNW_beg_ C40 for GM, C97 for GL, C23 for SOL, and C49 for PL. In RUN2, *triceps surae* push‐off activity remained unchanged, while PL push‐off activity decreased gradually. During RLD_tr_, *triceps surae* push‐off activity remained unchanged in either run. In contrast, PL push‐off activity increased in the two runs. Yet, no completion of this reloading adjustment could be identified, as PL push‐off activity was already not significantly different from RLD_end_ by RLD_tr_ $\bar{C}$1.

Furthermore, a Cycle effect was found for hamstring push‐off activity (ST‐SM: *F*_(405, 28896)_ = 23, BF: *F*_(405, 28896)_ = 12; *p* < 0.001) (Figure 5e,f). It increased gradually during UNW_tr_, showing a transient excessive increase between UNW_tr_ $\bar{C}$7‐UNW_beg_ C9 for ST‐SM (ES = 0.27 ± 0.05) and BF (ES = 0.34 ± 0.03). Conversely, it decreased progressively during RLD_tr_. Although a Cycle effect was found for the TA (*F*_(405, 25832)_ = 3, *p* < 0.001) and *quadriceps* muscles (VM: *F*_(405, 28896)_ = 1, VL: *F*_(405, 28896)_ = 2 and RF: *F*_(405, 28084)_ = 1; *p* < 0.001), post hoc analyses revealed only sporadic adjustments during either UNW_tr_ or RLD_tr_.

#### 3.2.4 Correlations Between RMS EMG and Mean Vertical Force

**FIGURE 6:**
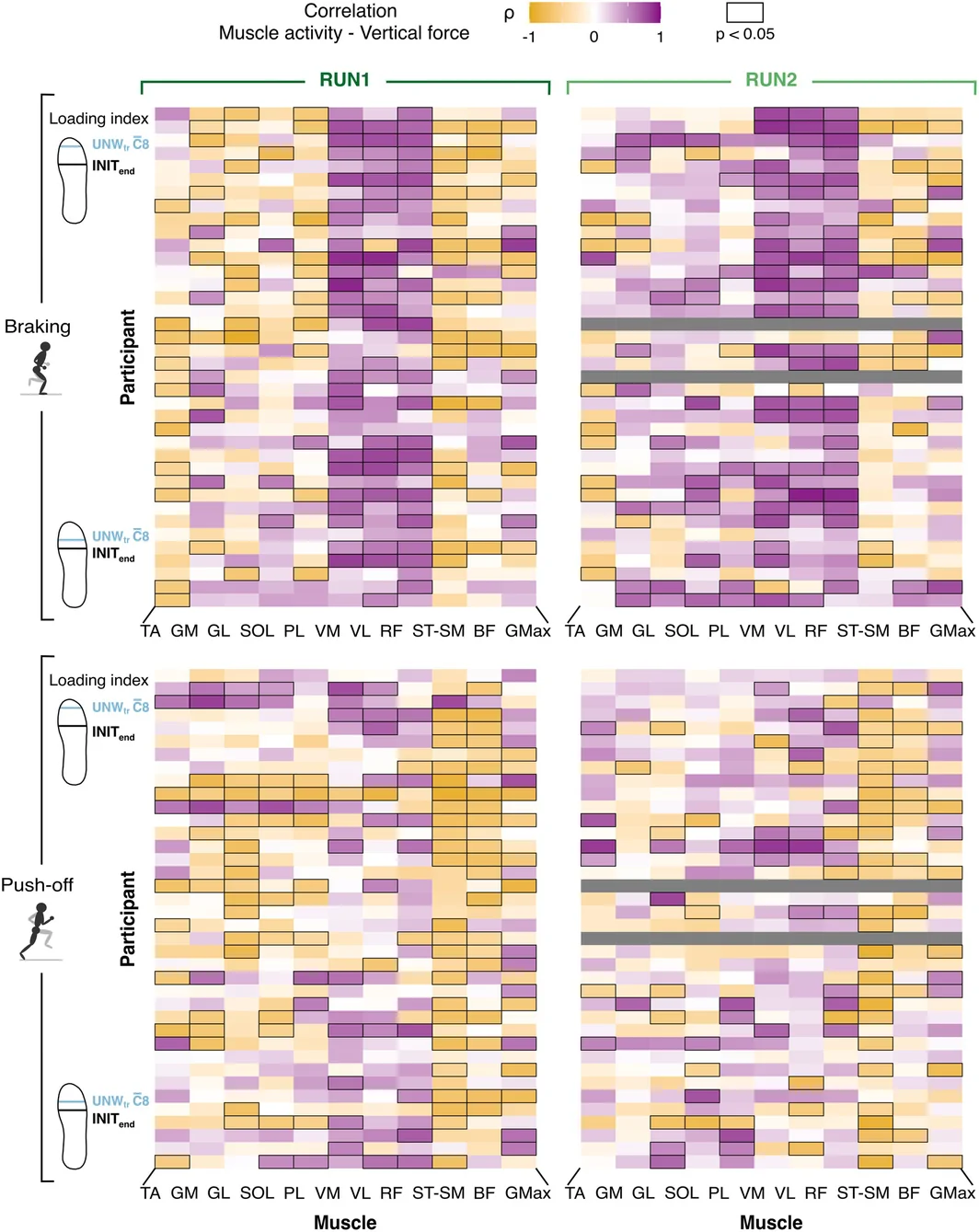
**Individual correlations between mean EMG activity and vertical force during** **UNW**_**tr**_. Spearman's correlation coefficients (*ρ*) between mean EMG activity and vertical force are shown during the braking and the push‐off phases, separately for each run (RUN1 in dark green, RUN2 in light green). Each row corresponds to a muscle, and each column to a participant. Colors indicate the direction of the correlations: Orange for negative, purple for positive, and white for null correlations. Color intensity indicates the strength of the correlations. Participants are ordered according to their adjustment in the loading index, from greatest adjustment (bottom) to the smallest adjustment (top). Statistically significant correlations (*p* < 0.05) are framed in black.

Spearman's correlations between adjustments in RMS EMG and in mean vertical force during UNW_tr_ are presented in Figure 6, for each participant separately.

During the braking phase, significant positive correlations predominated for the *quadriceps* in RUN1 (VM and RF: *χ* ^2^ = 22, VL: *χ* ^2^ = 17; *p* < 0.001) and RUN2 (VM: *χ* ^2^ = 18, VL: *χ* ^2^ = 15, RF: *χ* ^2^ = 22; *p* < 0.001). They also predominated for GM (*χ* ^2^ = 5, *p* < 0.05) and SOL (*χ* ^2^ = 6, *p* < 0.05) in RUN2. Conversely, significant negative correlations predominated for the hamstrings (STSM: *χ* ^2^ = 16, BF: *χ* ^2^ = 11; *p* < 0.001) and some lower leg muscles (TA: *χ* ^2^ = 10, GL: *χ* ^2^ = 8, PL: *χ* ^2^ = 7; *p* < 0.01) in RUN1, but only for TA (*χ* ^2^ = 6, *p* < 0.05) in RUN2.

During the push‐off phase, significant positive correlations between RMS EMG and mean vertical force predominated for VM (*χ* ^2^ = 5, *p* < 0.05) in RUN1. Yet, significant negative correlations predominated for the hamstrings in RUN1 (ST‐SM: *χ* ^2^ = 20, BF: *χ* ^2^ = 18, *p* < 0.001) and RUN2 (ST‐SM; *χ* ^2^ = 23, *p* < 0.001; BF: *χ* ^2^ = 9, *p* < 0.01), as well as for GL in RUN1 (*χ* ^2^ = 6, *p* < 0.05).

### 3.3 Neural Adjustments Based on Muscle Synergies

**FIGURE 7:**
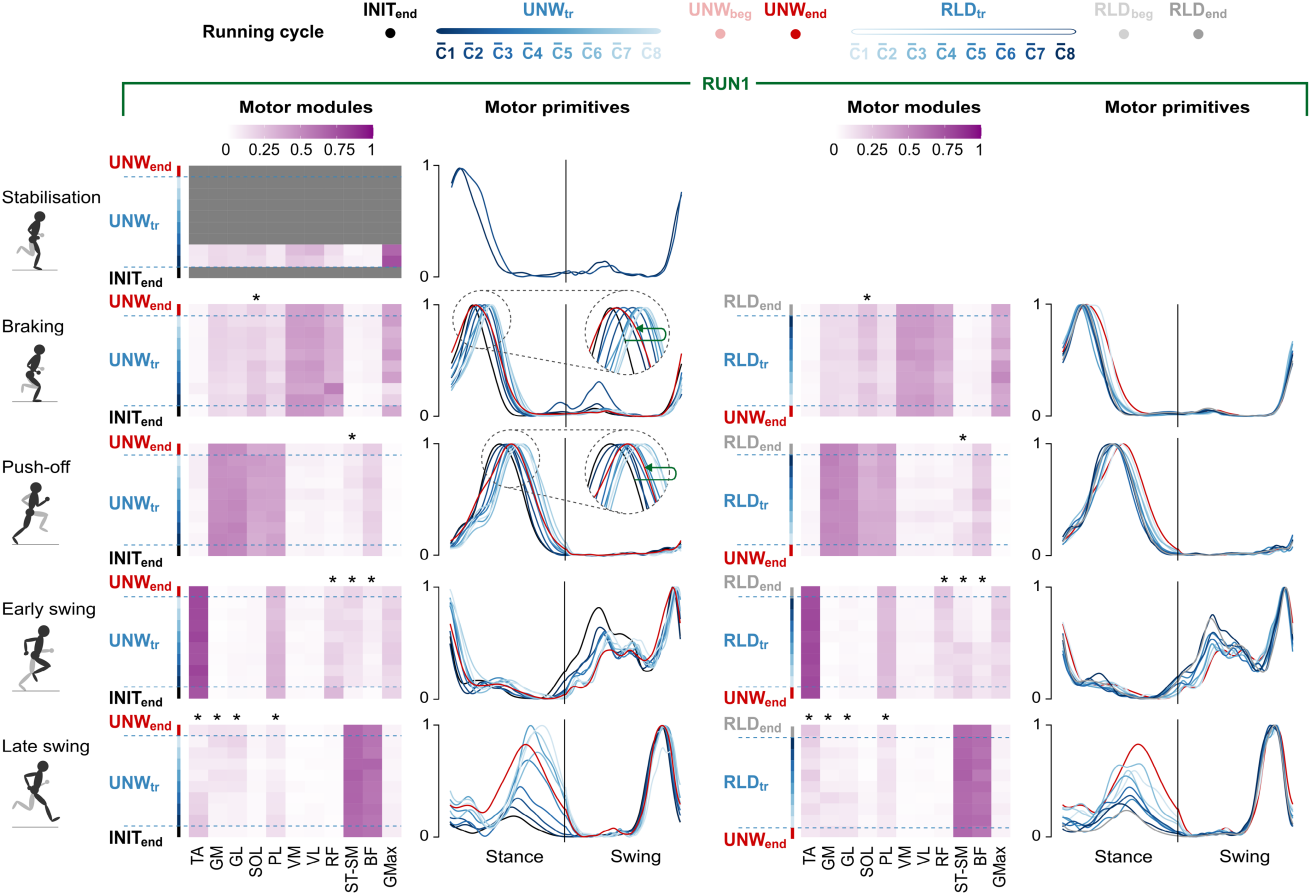
**Dynamics of muscle synergy adjustments in** **RUN1**. Motor modules and motor primitives are shown for each running cycle of the transitions (UNW_tr_ $\bar{C}$1–$\bar{C}$8 and RLD_tr_ $\bar{C}$1–$\bar{C}$8, in blue), and averaged over the 30 running cycles constituting the late stable phases (INIT_end_ in black, UNW_end_ in red and RLD_end_ in gray). For the motor modules, each row corresponds to a running cycle and each column to a muscle. Muscle contributions are color‐coded from white (no contribution) to dark purple (maximal contribution, equal to 1). Asterisks indicate the muscle contributions which showed a significant adjustment. For the motor primitives, the *x*‐axis separates the stance and swing phases, normalized to the same number of time points, while the *y*‐axis represents the normalized amplitude.

The muscle synergies extracted in RUN1 are presented in Figure 7. Due to minor differences, the synergies extracted in RUN2 are shown in Figure S1.

#### 3.3.1 Functional Role of Extracted Muscle Synergies

Four muscle synergies were identified across all running cycles in the two runs. In RUN1 only, a fifth muscle synergy was observed at UNW_tr_ $\bar{C}$1 and UNW_tr_ $\bar{C}$2. These synergies effectively reconstructed the pre‐processed EMG signals (RUN1, *R* ^2^ = 0.92 ± 0.004; RUN2, *R* ^2^ = 0.92 ± 0.004) and were functionally associated with distinct phases of the running cycle.

Among the four muscle synergies consistently identified, the braking synergy exhibited a major contribution from the hip and knee extensors muscles (GMax, VM, VL, RF). The push‐off synergy involved the ankle plantarflexors (GM, GL, SOL, PL). The early swing synergy recruited the ankle dorsiflexors (TA, PL). The late swing synergy showed a major contribution from the hamstrings (ST‐SM, BF). It corresponded to the late swing phase at INIT_end_ and RLD_end_, hence its designation. However, it showed an additional activation during the stance phase at UNW_end_, which increased progressively during UNW_tr_ and decreased progressively during RLD_tr_.

**FIGURE 8:**
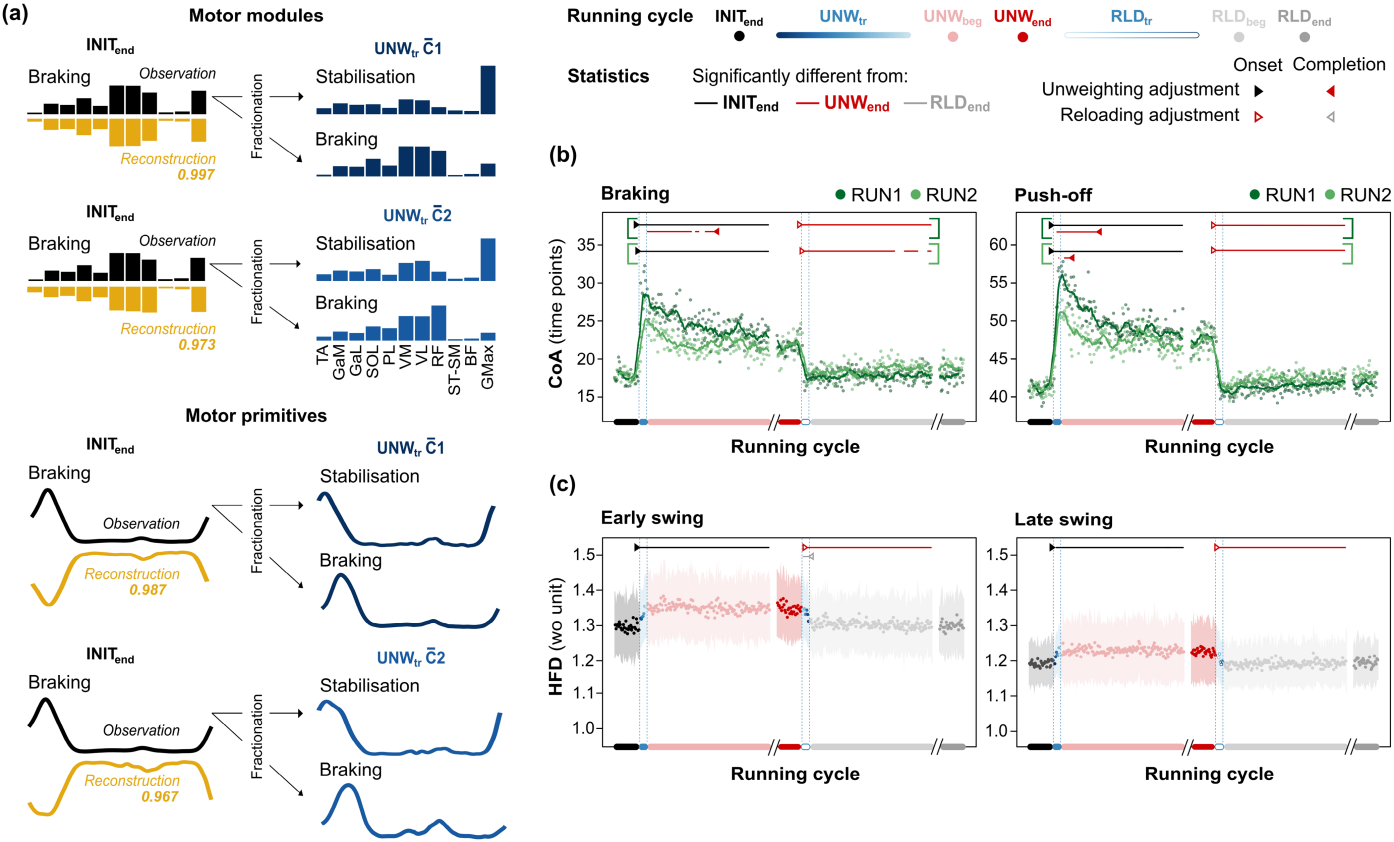
**Quantitative assessment of muscle synergy adjustments.** (a) Fractionation of the braking synergy. A linear combination of the motor modules (motor primitives) from the stabilization and braking synergies observed at UNW_tr_ $\bar{C}$1 and UNW_tr_ $\bar{C}$2 in RUN1 accurately reconstructed those observed at INIT_end_ in RUN1. The cosine similarity between the observed and reconstructed motor modules (primitives) is indicated in italics. (b) The center of activity (CoA) of the stance primitives (in time points) is shown for each cycle during the transitions (UNW_tr_ and RLD_tr_, in blue), early stable phases (UNW_beg_ in light red, RLD_beg_ in light gray) and late stable phases (INIT_end_ in black, UNW_end_ in red and RLD_end_ in gray). It is presented separately for RUN1 (dark green) and RUN2 (light green), due to a Cycle × Run interaction, and smoothed with a 10‐cycle moving average. (c) The Higuchi's fractal dimension (HFD) of swing primitives (mean ± SD, without unit) is shown for the same cycles as in (b), but averaged over both runs. In panels (b, c), horizontal bars indicate cycles that differ significantly from INIT_end_ (black), UNW_end_ (red), and RLD_end_ (gray). Arrowheads indicate the onset and completion of the adjustments.

The fifth synergy, observed exclusively at UNW_tr_ $\bar{C}$1 and UNW_tr_ $\bar{C}$2 in RUN1, was characterized by an almost exclusive contribution from the GMax. This synergy was activated just before the braking synergy and is hereafter referred to as the ‘stabilization synergy’. Importantly, a linear combination of the stabilization and braking synergies observed at UNW_tr_ $\bar{C}$1 and UNW_tr_ $\bar{C}$2 in RUN1 accurately reconstructed the braking synergy observed at INIT_end_, in terms of both motor modules and motor primitives. This indicates that this stabilization synergy emerged from the fractionation of the braking synergy observed at INIT_end_ (Figure 8a).

#### 3.3.2 Muscle Contributions

A Cycle effect was found for the cosine similarity of the four consistent synergies (braking: *F*_(405, 28248)_ = 5, push‐off: *F*_(405, 27057)_ = 14, early swing: *F*_(405, 21636)_ = 9, and late swing: *F*_(405, 24725)_ = 16; *p* < 0.001). The cosine similarity remained above 0.8 across all cycles in the two runs, indicating that motor modules were highly similar to those observed at INIT_end_ in RUN1. However, it decreased progressively during UNW_tr_ and increased during RLD_tr_. This suggests that subtle adjustments in motor modules occurred. In particular, during UNW_tr_, the contribution of the SOL to the braking synergy declined gradually; the contribution of the RF to the early swing synergy also decreased, while that of the hamstrings increased; the contribution of the TA and PL to the late swing synergy decreased, while that of the *gastrocnemii* increased (Figure 7).

#### 3.3.3 Motor Primitives Timing

The CoA of stance synergies exhibited a Cycle effect (braking: *F*_(405, 28248)_ = 12, push‐off: *F*_(405, 27057)_ = 19; *p* < 0.001), and a Cycle × Run interaction (braking: *F*_(405, 28248)_ = 2, push‐off: *F*_(405, 27057)_ = 2; *p* < 0.001) (Figure 8b). During UNW_tr_, the CoA of stance synergies increased progressively (the motor primitives shifted later in the running cycle), though the adjustment dynamics differed between runs. In RUN1, it showed a transient excessive increase: for the braking synergy, between UNW_tr_ $\bar{C}$1‐UNW_beg_ $\bar{C}$73 (ES = 0.6 ± 0.2); and for the push‐off synergy, between UNW_tr_ $\bar{C}$4‐UNW_beg_ C43 (ES = 0.5 ± 0.2). In RUN2, it no longer exhibited such overshoot. Conversely, during RLD_tr_, the CoA of the stance synergies decreased progressively in both runs.

There was also a Cycle effect for the CoA of swing synergies (early swing: *F*_(405, 21636)_ = 13, late swing: *F*_(405, 24725)_ = 14; *p* < 0.001). The CoA of the early swing synergy increased gradually from UNW_tr_ $\bar{C}$1 to UNW_tr_ $\bar{C}$4, and decreased from RLD_tr_ $\bar{C}$3. Conversely, the CoA of the late swing synergy decreased progressively from UNW_tr_ $\bar{C}$2 to UNW_tr_ $\bar{C}$3, and increased from RLD_tr_ $\bar{C}$3 to RLD_tr_ $\bar{C}$7.

#### 3.3.4 Motor Primitives Duration

We identified a Cycle effect for the FWHM of late swing synergy (*F*_(405, 24725)_ = 14, *p* < 0.001). It widened progressively from UNW_tr_ $\bar{C}$1 to UNW_tr_ $\bar{C}$2, and narrowed from RLD_tr_ $\bar{C}$2 to RLD_tr_ $\bar{C}$6.

In addition, there was a Cycle effect for the FWHM of stance synergies (braking: *F*_(405, 28248)_ = 1, *p* < 0.001; push‐off: *F*_(405, 27057)_ = 1, *p* < 0.05). Although the FWHM of the braking synergy was slightly higher at UNW_end_ than at INIT_end_ (*p* < 0.001, ES = 0.22), post hoc analysis revealed only sporadic adjustments during UNW_tr_ and RLD_tr_.

#### 3.3.5 Motor Primitive Complexity

A Cycle effect was observed for the HFD of the swing synergies (early swing: *F*_(405, 21636)_ = 5, late swing: *F*_(405, 24725)_ = 5; *p* < 0.001) (Figure 8c). During UNW_tr_, the HFD of swing synergies increased progressively. This adjustment showed no completion, as it did not differ significantly from UNW_end_ by UNW_tr_ $\bar{C}$1. Conversely, it declined gradually during RLD_tr_.

Finally, the HFD of stance synergies showed a Cycle effect (braking: *F*_(405, 28248)_ = 2, push‐off: *F*_(405, 27057)_ = 1; *p* < 0.001), as well as a Cycle × Run interaction (braking: *F*_(405, 28248)_ = 1, *p* < 0.05; push‐off: *F*_(405, 27057)_ = 1, *p* < 0.001). Although the HFD of the braking synergy was slightly higher at UNW_end_ than at INIT_end_ and RLD_end_ (*p* < 0.001, ES = 0.28 in the two runs), post hoc analyses showed no adjustment during the transitions.

## 4 Discussion

The aim of this study was to examine how neural adjustments unfold during progressive transitions to unweighted and reloaded running (UNW_tr_ and RLD_tr_) on a lower body positive pressure treadmill (LBPPT), and to assess the repeatability of their dynamics across two successive exposures (RUN1, RUN2). Consistent with our first hypothesis, some neural adjustments were completed by the end of UNW_tr_ in both runs, but others remained incomplete by the end of UNW_tr_ in RUN1. In line with our second hypothesis, these neural adjustments were achieved earlier in RUN2. Yet, contrary to our third hypothesis, the neural adjustments did not exhibit the same dynamics during UNW_tr_ and RLD_tr_. Notably, the adjustments that were incomplete by the end of UNW_tr_ were fully completed by the end of RLD_tr_, even in RUN1.

### 4.1 Completed Adjustments by the End of UNW_tr_ in Both Runs

Supporting central adjustments, the preactivation of the *tibialis anterior*, and its contribution to the late swing synergy, decreased progressively during UNW_tr_. This reflects a shift toward a forefoot strike pattern, previously reported during the stable phase of unweighted running [29]. In contrast, the preactivation of the g*astrocnemii* remained unchanged, despite the earlier observation of a transient increase [9]. Such discrepancy is attributed to the smaller increase in swing time (about half) observed in the present study due to differences in LBPPT design.

In the subsequent braking phase, the EMG activity of the *soleus* and *quadriceps* decreased gradually during UNW_tr_. This may stem from decreases in both central activity and the stretch‐reflex response. The latter has been shown to be attenuated in the *vasti* when running at 60% body weight on a LBPPT [4]. It is worth noting that the reduction in *quadriceps* braking activity was more pronounced than in the other muscles and was positively correlated with the reduction in vertical braking force in most participants (VM: *r* = 0.58 ± 0.15, VL: 0.58 ± 0.12; RF: 0.56 ± 0.13). These findings may be specific to the LBPPT because the vertical pelvic support likely reduced stretch velocity more in the proximal extensor muscles than in the distal ones. In any case, they agree with simulation studies reporting that the *quadriceps*, rather than the *soleus*, mainly contribute to the braking phase during running [30].

In contrast, both the braking and push‐off EMG activity of the hamstrings increased gradually during UNW_tr_. This may have contributed to the greater backward excursion of the swing limb observed in most participants. It might also have accelerated knee flexion during the early swing phase, as a compensatory mechanism to counteract the slowing of stride frequency [7]. Interestingly, this increase in the braking and push‐off activity of the hamstrings did not manifest in a greater contribution of this muscle group to the braking or push‐off synergies. Rather, it resulted in an additional activation burst of the late swing primitive during the stance phase. Researchers have proposed that the central nervous system does not reconfigure the “building blocks” for each task or condition but flexibly recruits them, a strategy that is thought to be less computationally costly [31].

Finally, the complexity of the early and late swing primitives increased progressively during UNW_tr_, indicating greater flexibility of locomotor control during the swing phase [8]. In other words, runners may have exerted less stride‐to‐stride control. This may appear surprising, since the swing leg does not benefit from body weight support on the LBPPT [4]. Yet, such greater flexibility was likely enabled by pelvic vertical support, which ensured body stability despite alterations in the swing leg's trajectory and velocity.

### 4.2 Incomplete Adjustments at the End of UNW_tr_ in RUN1

Hamstring preactivation decreased progressively, probably due to the increased passive retraction of the swing‐leg associated with a longer swing time [32]. Importantly, this decrease reached only half of its final magnitude by the end of UNW_tr_ and continued during UNW_beg_. Because muscle preactivation relies on feedforward control, the delayed completion of this adjustment likely reflects the time required to update internal models, based on the mismatch between predicted and actual swing leg dynamics [33]. Such a process may have been further delayed by the lack of visual feedback from the lower limbs when running on a LBPPT, which encourages the adoption of a ‘default pattern of motor commands’ [34]. The transient insufficient decrease in hamstring preactivation may have contributed to the transient excessive decrease in *vasti* preactivation through non‐optimized feedforward control.

The braking activity of the *gastrocnemii* and *peroneus longus* muscles remained unchanged during UNW_tr_, before decreasing progressively during UNW_beg_ in RUN1. This absence of adjustment during UNW_tr_ is likely due to the observed exaggerated shift toward a forefoot strike pattern, which may have increased the sensory constraints. Interestingly, the current study revealed that runners who exhibited the most pronounced increase in the loading index (i.e., the most pronounced shift toward a forefoot strike pattern) were more likely to show an increase in the braking activity of shank muscles (GM, GL, PL) during UNW_tr_. This finding aligns with previous studies showing that forefoot runners exhibit higher *gastrocnemii* EMG activity than rearfoot runners during the stance phase [35]. The subsequent decrease in *gastrocnemii* and *peroneus longus* braking activity during UNW_beg_ is likely due to the loading index stabilizing at a lower value than that observed at the end of UNW_tr_. This suggests a partial normalization of the foot strike pattern.

In the push‐off phase, the EMG activity of the *triceps surae* and *peroneus longus* muscles increased during UNW_tr_, before decreasing gradually during UNW_beg_ in RUN1. Interestingly, this initial increase in *triceps surae* and *peroneus longus* EMG activity was not accompanied by a change in their contribution to the push‐off synergy. Instead, these muscles showed an increased contribution to the late swing synergy, which also involves the hamstrings. This highlights a co‐activation of the *triceps surae*, *peroneus longus*, and hamstring muscles (i.e., a greater functional coupling of the posterior chain muscles) during the push‐off phase, likely to maintain the imposed running speed despite a transient excessive decrease in stance time, largely driven by a transient excessive decrease in push‐off time. The subsequent reduction in their push‐off activity during UNW_beg_ is attributed to a gradual attenuation in temporal constraints, as stance time stabilized at a lower value than that observed at the end of UNW_tr_.

Finally, stance motor primitives shifted later in the running cycle during UNW_tr_ in RUN1, as reported in mice and humans undergoing external perturbations [20]. This shift exceeded its final magnitude by the end of UNW_tr_. It was nearly twice as large as that expected from the stance phase normalization alone, strongly suggesting that it resulted from the peaked temporal and sensory constraints.

### 4.3 Transient Adjustments During UNW_tr_ in RUN1

An unexpected but interesting observation is the recruitment of a stabilization synergy during the first two running cycles of UNW_tr_ in RUN1. This additional synergy resulted from the fractionation of the usual braking synergy. It corresponded to the recruitment of the *gluteus maximus* at the very beginning of the stance phase, before the recruitment of the other extensor muscles. It was observed in around 80% of the participants, indicating that it played a genuine functional role rather than being a mere computational artifact. Notably, it may have stabilized the hip joint at the onset of unweighting transition since runners tended to lean forward onto the LBPPT chamber. The fractionation of muscle synergies has been poorly documented. It may be a biomarker of the central nervous system maturity and integrity. In this sense, it has been found during child‐to‐adult development of stepping and running [26], and relates to post‐stroke duration [25]. Our findings suggest that the fractionation of muscle synergies may also reflect difficulty in adjusting to novel or demanding conditions.

### 4.4 Faster Adjustments During UNW_tr_ in RUN2

As expected, the adjustments that were incomplete at the end of UNW_tr_ in RUN1 were completed earlier in RUN2.

Notably, hamstring preactivation decreased during UNW_tr_ in RUN2, without exhibiting the transient insufficient decrease observed in RUN1. This suggests that a single exposure enabled the feedforward control to account more quickly for the increased passive retraction of the swing leg, despite the continued absence of visual feedback from the lower limbs. Consequently, *vasti* preactivation no longer exhibited a transient excessive decrease. Interestingly, the preactivation of the other muscles did not change. The support provided by the LBPPT chamber at the pelvis may have reduced muscle stretch velocity more at the proximal than at the distal level. This assumption is supported by the fact that peak knee flexion decreased by a greater amount than peak angle flexion during the stance phase [36]. In addition to this device‐specific explanation, neural adjustments may preferentially emerge near to the point at which the perturbation is applied (near to the center of mass). Indeed, in the case of ground‐related perturbations, compensatory responses have predominantly been reported at the ankle joint [37, 38]. Besides, previous studies on postural control have demonstrated that the CNS's strategy strongly depends on the point of application of the perturbation [39].

The braking activity of the *gastrocnemii* and *peroneus longus* decreased immediately, and their adjustment was already completed by the end of UNW_tr_ in RUN2. This indicates that, upon re‐exposure, runners anticipated the shift toward a forefoot strike pattern as less destabilizing for the ankle. First, the shift toward a forefoot strike pattern was less pronounced during UNW_tr_ in RUN2, as evidenced by the smaller increase in the loading index. Second, runners may have become accustomed to the altered strike pattern and the increased demand for ankle stabilization it entails. Along this line, previous studies have reported that *peroneus longus* activity is reduced following repeated exposure to rapid ankle inversion while standing [40].

During the push‐off phase, the EMG activity of the *triceps surae* and *peroneus longus* no longer increased during UNW_tr_ in RUN2. This is likely because stance time no longer showed a transient excessive decrease. Furthermore, the stance motor primitives no longer exhibited a transient excessive shift within the running cycle. This aligns with the fact that the temporal constraints, although still elevated, were no longer transiently exacerbated.

In addition, the stabilization (*gluteus maximus*) muscle synergy recruited during the early braking phase in the first two cycles of UNWtr in RUN1 no longer occurred. This simplification of locomotor control upon re‐exposure is consistent with a recent study reporting that the extra muscle synergies recruited during the early exposures to a gait‐slip perturbation, through the fractionation of pre‐existing synergies, disappear with repeated exposure [15]. More broadly, this supports the idea that both the fractionation and merging of muscle synergies underlie motor learning [25, 26].

Together, these results offer a novel perspective on those previously published [5, 8]. Although a similar neuromuscular state was ultimately achieved during the stable phase of unweighted running in both runs, the dynamics leading to this state during the transition phase differed markedly. The final state emerged gradually, through suboptimal and sometimes transient adjustments during UNW_tr_ in RUN1. Yet, it was reinstated rapidly during UNW_tr_ in RUN2, providing strong evidence of ‘savings’. This phenomenon, whereby the nervous system recalls previously learned motor patterns, has been well‐documented during running on a split‐belt treadmill [13, 14]. Notably, runners demonstrated savings during UNW_tr_ in RUN2 despite having experienced the opposite perturbation during RLD_tr_ in RUN1. This finding is consistent with studies suggesting that savings can persist even when competing adjustments to an opposite perturbation occur between exposures [13].

### 4.5 Distinct Adjustments During UNW_tr_ and RLD_tr_

The adjustments that were incomplete by the end of UNW_tr_ in RUN1 were fully completed by the end of RLD_tr_ in RUN1.

One possible explanation is that RLD_tr_ re‐introduced a familiar sensorimotor context resulting from our lifelong exposure to normogravity, whereas UNW_tr_ presented an unusual sensorimotor context. The latter was likely marked by reduced cutaneous afferents from plantar sole mechanoreceptors, as reported when standing at 40% body weight [41]. It was also likely characterized by reduced proprioceptive muscle afferents. Indeed, the vertical displacement of the center of mass decreased by a greater amount than the braking time when running at 60% body weight [4, 5], suggesting a reduction in muscle stretch velocity. Importantly, the reliance on reduced cutaneous and proprioceptive afferents may have been amplified by the absence of visual feedback from the lower limbs when running on the LBPPT, thereby making the sensorimotor context further challenging.

Another possible explanation lies in the order of the transitions: runners always experienced UNW_tr_ before RLD_tr_. In this sense, previous studies have shown that adjustments to visuomotor or locomotor perturbations can generalize to similar perturbations of different magnitude [14, 42, 43]. This suggests that the central nervous system learns how to adjust rather than storing a permanent memory of the adjusted pattern. Accordingly, the initial exposure to UNW_tr_ in RUN1 may have facilitated the adjustments not only during UNW_tr_ in RUN2 but also during RLD_tr_ in RUN1.

Finally, the progressive increase in EMG activity during RLD_tr_ may have been easier to control than the progressive decrease during UNW_tr_. While running involves polyarticular movements, it is well known that increasing muscle force is generally more accurately and less variably controlled than decreasing it in simpler, monoarticular movements [44].

### 4.6 Limits

This study did not include kinematic measurements that would have confirmed the presumed changes in swing leg's trajectory or velocity. Such measurements are challenging on the LBPPT. Indeed, the curvature and thickness of its transparent chamber causes optical distortion, which interferes with standard video‐based motion capture. Moreover, all participants underwent UNW_tr_ before RLD_tr_. This fixed order prevents a clear dissociation between the effects of the sensorimotor context (unusual versus familiar) and of prior exposure on the dynamics of adjustments during RLD_tr_.

## 5 Conclusion

The transition to unweighted running presented a new sensorimotor context, resulting in some incomplete or transient adjustments upon first exposure. However, such adjustments occurred more rapidly upon re‐exposure, providing strong evidence of savings. In contrast, the transition to reloaded running reinstated a familiar sensorimotor context, enabling complete adjustments from first exposure. Overall, this study showed that the dynamics of neural adjustments—that is, how they unfold over time—constitute a more sensitive marker of locomotor adaptability than their steady‐state expression.

## 6 Perspectives

Progressive transitions to unweighted and reloaded running provide a valuable framework for assessing locomotor adaptability. In clinical settings, this approach may reveal sensorimotor impairments, especially proprioceptive impairments. As a result, it may aid in identifying individuals at higher risk of falls, allowing for early and personalized interventions. Beyond assessment, progressive transitions to unweighted and reloaded running may be used to maintain, restore, or even enhance locomotor adaptability by exposing patients to repeated sensorimotor challenges under safe conditions. That said, clinicians should exercise caution when implementing unweighting transitions, whether for assessment or rehabilitation purposes, in patients with metatarsal or hamstring vulnerabilities. Indeed, the shift toward a forefoot strike pattern and the increase in hamstring braking and push‐off activity may place additional strain on these structures.

## Funding

The study was supported by the Centre National d'Études Spatiales, Département des Sciences de la Vie et de Médecine Spatiale (Grant DAR 4800001107).

## Ethics Statement

The study was approved by the National Ethics Committee for Research in Sports Sciences (reference no. CERSTAPS IRB00012476‐2021‐31‐03‐96).

## Consent

All participants gave written informed consent prior to the experiment.

## Conflicts of Interest

The authors declare no conflicts of interest.

## Supporting information


**Figure S1:** **Dynamics of muscle synergies adjustments in RUN2**. Motor modules and motor primitives are shown for each running cycle of the transitions (UNW_tr_ $\bar{C}$1–$\bar{C}$8 and RLD_tr_ $\bar{C}$1–$\bar{C}$8, in blue), and averaged over the 30 running cycles constituting the late stable phases (INIT_end_ in black, UNW_end_ in red and RLD_end_ in gray). For the motor modules, each row corresponds to a running cycle and each column to a muscle. Muscle contributions are color‐coded from white (no contribution) to dark purple (maximal contribution, equal to 1). Asterisks indicate the muscle contributions which showed a significant adjustment. For the motor primitives, the *x*‐axis separates the stance and flight phases, normalized to the same number of time points, while the *y*‐axis represents the normalized amplitude.

## Data Availability

Data are available upon reasonable request from the corresponding author.
