# Supplementary material for: Dynamics of Neural Adjustments During Progressive Transitions to Unweighted and Reloaded Running
Source: Scand J Med Sci Sports. 2026 Aug 24;36(9):e70363. doi: 10.1111/sms.70363 (PMC13502570; doi:10.1111/sms.70363)
Supplement: Supplementary file 1 [file SMS-36-e70363-s001.docx]

**Supplementary Figure 1:** **Dynamics of muscle synergies adjustments in RUN2**

Motor modules and motor primitives are shown for each running cycle of the transitions (UNW_tr_ $\bar{C}$1-$\bar{C}$8 and RLD_tr_ $\bar{C}$1-$\bar{C}$8, in blue), and averaged over the 30 running cycles constituting the late stable phases (INIT_end_ in black, UNW_end_ in red and RLD_end_ in grey). For the motor modules, each row corresponds to a running cycle and each column to a muscle. Muscle contributions are color-coded from white (no contribution) to dark purple (maximal contribution, equal to 1)**.** Asterisks indicate the muscle contributions which showed a significant adjustment. For the motor primitives, the x-axis separates the stance and flight phases, normalized to the same number of time points, while the y-axis represents the normalized amplitude.
